# A filter approach for feature selection in classification: application to automatic atrial fibrillation detection in electrocardiogram recordings

**DOI:** 10.1186/s12911-021-01427-8

**Published:** 2021-05-04

**Authors:** Pierre Michel, Nicolas Ngo, Jean-François Pons, Stéphane Delliaux, Roch Giorgi

**Affiliations:** 1 CNRS, EHESS, Centrale Marseille, AMSE, Aix-Marseille Univ, Marseille, France; 2 INSERM, IRD, SESSTIM, Sciences Economiques & Sociales de la Santé & Traitement de l’Information Médicale, Aix Marseille Univ, Marseille, France; 3 WitMonki SAS, Marseille, France; 4 INSERM, INRAE, C2VN, Aix Marseille Univ, Marseille, France; 5 Hôpital Nord, Service des Explorations Fonctionnelles Respiratoires, Pôle cardiovasculaire, APHM, Marseille, France; 6 APHM, INSERM, IRD, Sciences Economiques & Sociales de la Sante & Traitement de l’Information Médicale (SESSTIM), Hop Timone, Biostatistique et Technologies de l’Information et de la Communication (BioSTIC), Aix Marseille Univ, Marseille, France

**Keywords:** $\gamma$-metric, Machine learning, Feature selection, Classification, Clinical decision making, Atrial fibrillation detection

## Abstract

**Background:**

In high-dimensional data analysis, the complexity of predictive models can be reduced by selecting the most relevant features, which is crucial to reduce data noise and increase model accuracy and interpretability. Thus, in the field of clinical decision making, only the most relevant features from a set of medical descriptors should be considered when determining whether a patient is healthy or not. This statistical approach known as feature selection can be performed through regression or classification, in a supervised or unsupervised manner. Several feature selection approaches using different mathematical concepts have been described in the literature. In the field of classification, a new approach has recently been proposed that uses the $\gamma$-metric, an index measuring separability between different classes in heart rhythm characterization. The present study proposes a filter approach for feature selection in classification using this $\gamma$-metric, and evaluates its application to automatic atrial fibrillation detection.

**Methods:**

The stability and prediction performance of the $\gamma$-metric feature selection approach was evaluated using the support vector machine model on two heart rhythm datasets, one extracted from the PhysioNet database and the other from the database of Marseille University Hospital Center, France (Timone Hospital). Both datasets contained electrocardiogram recordings grouped into two classes: normal sinus rhythm and atrial fibrillation. The performance of this feature selection approach was compared to that of three other approaches, with the first two based on the Random Forest technique and the other on receiver operating characteristic curve analysis.

**Results:**

The $\gamma$-metric approach showed satisfactory results, especially for models with a smaller number of features. For the training dataset, all prediction indicators were higher for our approach (accuracy greater than 99% for models with 5 to 17 features), as was stability (greater than 0.925 regardless of the number of features included in the model). For the validation dataset, the features selected with the $\gamma$-metric approach differed from those selected with the other approaches; sensitivity was higher for our approach, but other indicators were similar.

**Conclusion:**

This filter approach for feature selection in classification opens up new methodological avenues for atrial fibrillation detection using short electrocardiogram recordings.

**Supplementary Information:**

The online version contains supplementary material available at 10.1186/s12911-021-01427-8.

## Background

In statistics and high-dimensional data analysis, the scoring and ranking of individual features may be necessary for feature selection and dimension reduction [1]. Indeed, this approach reduces both the complexity of the model and the noise present in the data, which increases model accuracy and interpretability [2]. Feature selection is a data preprocessing technique that consists in generating the best possible feature subset through selecting the most relevant features and removing redundant or noisy ones. This technique speeds up classification (training and testing) and optimizes model accuracy (e.g., prediction error rate).

Typically, a feature selection algorithm includes four steps [3]: (1) subset generation, in which candidate feature subsets are selected based on certain search methods (e.g., exhaustive, random, or heuristic search method); (2) evaluation function computation, in which the relevance of the selected candidate subsets is assessed; (3) identification of a stopping criterion, in which the criterion for stopping the algorithm and returning the selected subset is specified; and, (4) result validation, in which the performance of the feature selection algorithm is tested on a distinct dataset.

In step 1, the feature selection algorithm can use several methods to search for candidate subsets. The most common are the exhaustive and heuristic search methods. These include greedy approaches whereby only local optimal choices are made in search space (for example, by adding or removing features sequentially through a forward and backward search) and a unidirectional search is conducted (the forward search starts with an empty feature set and the backward search with a full feature set). More complex heuristic search methods also exist. Among these is the “best-first search” approach [4], which is similar to the greedy approach, but differs from it in that it chooses the best neighbor subset among all evaluated ones. In this method, the user defines how many times the feature subset search is to be repeated. One should also mention the more computationally complex metaheuristic approaches, such as the ant-colony algorithm [5] and the genetic algorithm (GA) [6]. These two algorithms are known to provide satisfactory solutions to many optimization problems, including the travelling salesman problem. A recent paper comparing the performance of different state-of-the-art metaheuristic algorithms (including GA), found that these approaches may constitute good alternatives for the problem of parameter estimation in real world applications [7].

When the number of features *p* in a training dataset is too high (say $p > 100$), it becomes impossible to test all possible solutions, that is, the $2^p-1$ possible feature subsets. In this situation, feature selection becomes “NP-hard” [2]. Since the exhaustive search for solutions is not feasible, heuristic strategies must be considered, even though they can converge to local optima. One of these strategies is the forward search algorithm, which generates an initial solution with the most relevant features, evaluates this solution, and then assesses all feature subsets obtained by adding the most relevant feature from among the remaining ones.

In supervised learning (and especially in classification), the relevance of a feature is often assessed by quantifying its correlation with or dependence on a target feature *Y*, or by using consistency and separability indices or information theory-based metrics [8]. The feature selection method tries to find the best combination of features according to an evaluation function that quantifies the relevance of all features. Evaluation functions can be divided into five categories [9]: distance measures, information measures, dependence measures, consistency measures, and classifier error rate measures. Depending on the evaluation function, one can use either “wrapper” methods [10] to evaluate selected features based on the performance of a given classifier or “filter” methods to select features without employing a classifier.

Model complexity reduction in high-dimensional data analysis has many applications in the medical field. One such application is heart rhythm characterization, which is of clear clinical importance. At present, the main challenge for heart rhythm characterization is the automated detection of atrial fibrillation (AF). Indeed, AF, which is characterized by an irregular and often rapid heart rate, is associated with a five-fold increase in the risk of ischemic strokes [11]. It is currently the most common heart rhythm disorder and the second leading cause of mortality worldwide [12]. However, AF diagnosis is not obvious, especially in stroke patients, who often present with silent (asymptomatic) and mostly paroxysmal [13] forms. Fortunately, heart rhythm can be characterized by electrocardiogram (ECG), which is commonly used to explore electric cardiac activity and to detect arrhythmia. Automatic detection of AF is now a source of hope, especially with the development of connected medical devices.

In a recent study, Pons et al. introduced a new feature selection approach, which consists in selecting highly discriminant features from a set of features [14]. They examined whether this approach could be used for heart rhythm characterization using 1-min RR interval time series derived from ECG recordings. In heart rhythm analysis, an electrical cardiac cycle is traditionally divided into five waves denoted P, Q, R, S, and T, with R being the wave with maximal amplitude. An RR interval represents the time elapsed between two consecutive R waves that leads to one cardiac beat. Indeed, RR interval variability is often used as a marker of heart rhythm. The new approach introduced by Pons et al. is specifically aimed at improving discrimination between different heart rhythms: normal sinus rhythm (NSR) and AF. It uses a new evaluation function, the $\gamma$-metric, which is defined as the algebraic distance between classes. The approach showed good performance and improved classification accuracy by reducing both the number of features considered in the model and the necessary length of the time series, even in datasets with added noise or missing data. Not only are these preliminary results encouraging, but this approach could supersede current state-of-the-art feature selection approaches. However, Pons et al. [14] used a logistic regression as a classifier and an exhaustive approach for feature selection. Moreover, both their training and validation datasets were extracted from the same PhysioNet database available on the internet.

The present paper proposes a filter approach for feature selection in classification that uses the $\gamma$-metric introduced by Pons et al. as an evaluation function as well as the support vector machine (SVM) model to [15] solve the supervised classification problem. This approach was applied to AF detection using electrocardiogram recordings derived from two independent datasets (with the validation dataset containing real electrocardiogram data). The classification performance of the approach was evaluated.

## Methods

This section covers the following topics: the ECG datasets used for the analysis; the mode of computation of the $\gamma$-metric and its use as a filter approach for feature selection; the measure of consistency used to assess feature selection stability; the SVM model used as a classifier; and the strategy for feature selection used for AF detection considered as a classification task.

### Description of the datasets

Two ECG datasets were used in our study: a training dataset and a validation dataset.

#### Training dataset

The training dataset was extracted from the PhysioNet website [16], the open Massachusetts Institute of Technology-Beth Israel Hospital (MIT-BIH) NSR database (nsrdb), the MIT-BIH NSR RR interval database (nsr2db), and the MIT-BIH AF database (afdb). It contained 50, 028 1-min RR interval time series, 47, 128 of which corresponded to NSR rhythms and 2,900 to AF rhythms. No identifying information was used in the analysis.

#### Validation dataset

**Fig. 1:**
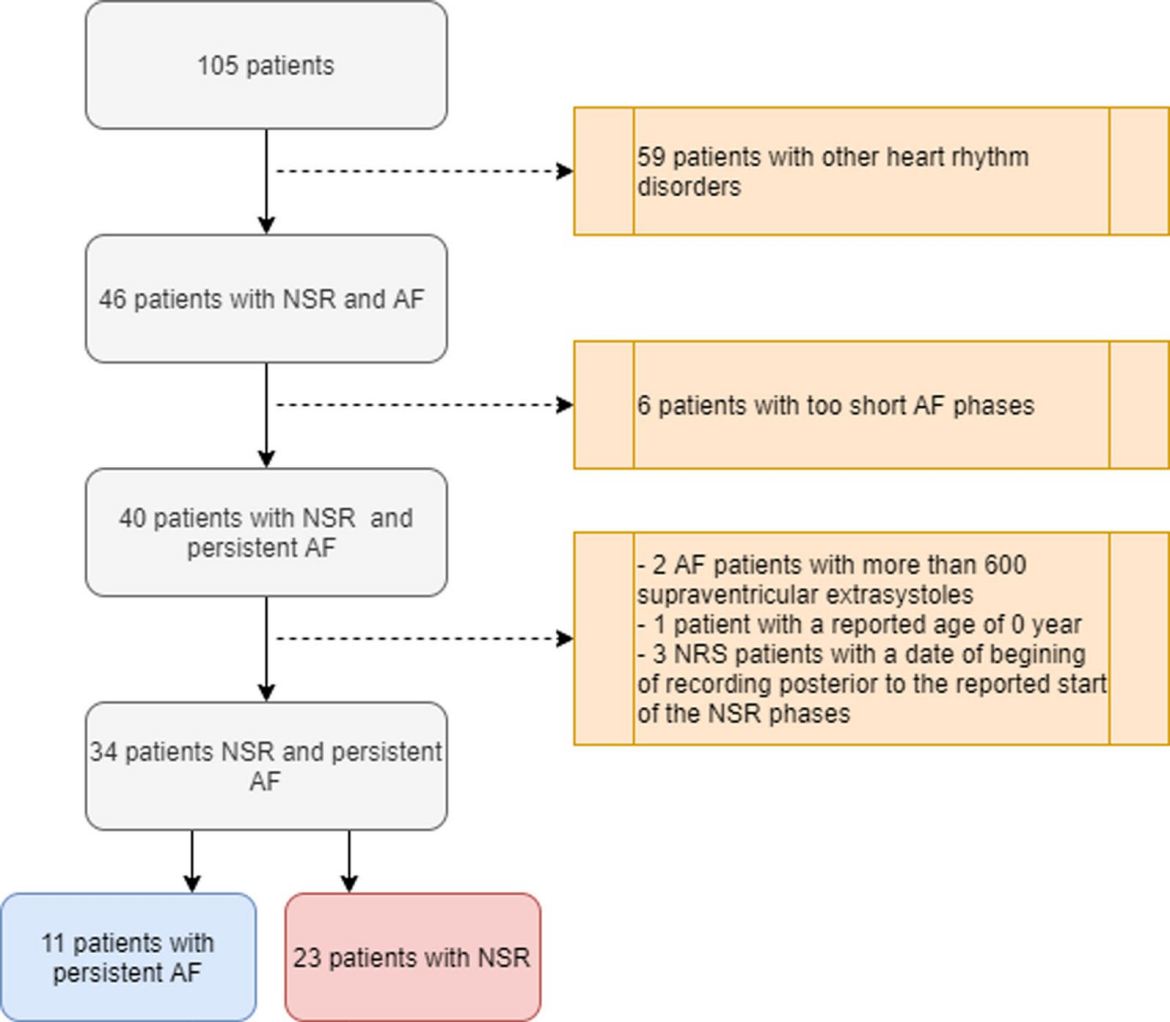
Flowchart of data preprocessing. Grey boxes show the number of patients retained at each stage. Orange boxes provide details on the reasons for patient exclusion. The red and blue boxes at the bottom show the number of NSR and AF patients retained for further analysis

The validation dataset was obtained from the Department of Cardiology and Rhythmology of Marseille University Hospital Center (Timone Hospital), France. A total of 105 patients undergoing continuous 24-hour Holter monitoring between 20 December 2016 and 26 February 2017 were considered for inclusion in the study. All ECG recordings were anonymized and no personal information was available except for age and gender. Preprocessing and quality check of RR interval time series consisted in tagging and excluding time series that contained misdetected R peaks (RR interval < 200ms) and/or undetected R peaks (RR interval > 3s). This procedure was aimed at ensuring the ECG signal quality (noise, signal interruption) of analyzed recordings and at assessing robustness of R peak detection against R peak detection algorithm limits. Premature ventricular and atrial contractions, which were present in the analyzed recordings of both AF and NSR patients, were not impacted by this preprocessing procedure. Patients with heart rhythm disorders other than AF were excluded from the analysis ($n = 59$). Of the remaining 46 patients, 6 AF patients were excluded because their AF episodes were deemed too short to be informative and analyzable (duration less than one minute), 2 AF patients due to unreliable annotations, and 4 NSR patients because their recordings contained inconsistent information (1 patient had a reported age of 0 years and 3 had time series with a recording start date posterior to the start date of the NSR episode). In the end, 34 patients were included the analysis, 11 of whom had AF (providing 11, 131 1-min RR interval time series) and 23 had NSR (yielding 30, 530 1-min RR interval time series). The flowchart presented in Fig. 1 summarizes the preprocessing procedure.

**Fig. 2:**
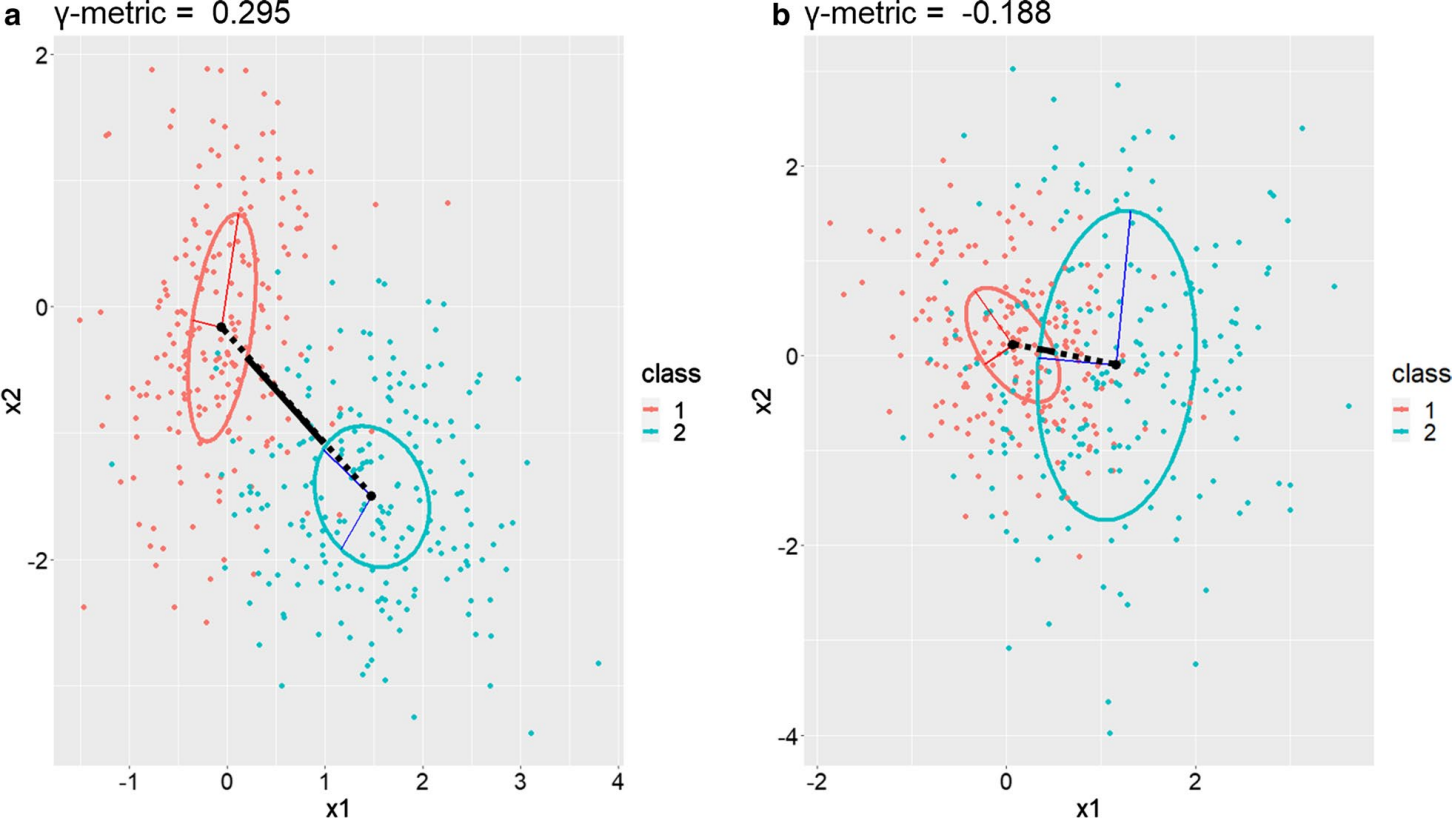
Ellipses obtained in the case of a two-dimensional space for two classes of observations. Each class *k* is generated using a different multivariate normal distribution $\mathcal {N}({\varvec{\mu }}_k, {\varvec{\Sigma }}_k)$. The first graph (**a**) is obtained considering $${\varvec{\mu }}_1 = t\left( \begin{array}{c} 0 \\ 0 \end{array}\right)$$, $${\varvec{\mu }}_2 = \left( \begin{array}{c} 1.5 \\ -1.5 \end{array}\right)$$, $${\varvec{\Sigma }}_1 = \left( \begin{array}{cc} 0.3 &{} 0.1 \\ 0.1 &{} 0.9 \end{array}\right)$$ and $${\varvec{\Sigma }}_2 = \left( \begin{array}{cc} 0.8 &{} 0 \\ 0 &{} 0.8 \end{array}\right)$$, resulting in a positive value of the $\gamma$-metric. The second graph (**b**) is obtained considering $${\varvec{\mu }}_1 =\left( \begin{array}{c} 0.1 \\ 0.1 \end{array}\right)$$, $${\varvec{\mu }}_2 = \left( \begin{array}{c} 1.1 \\ 0 \end{array}\right)$$, $${\varvec{\Sigma }}_1 = \left( \begin{array}{cc} 0.4 &{} -0.1 \\ -0.1 &{} 0.5 \end{array}\right)$$ and $${\varvec{\Sigma }}_2 = \left( \begin{array}{cc} 0.9 &{} 0 \\ 0 &{} 1.2 \end{array}\right)$$, resulting in a negative value of the $\gamma$-metric

For both datasets, 1-min RR interval time series were derived from ECG recording using a customized R-peak detector. RR intervals were expressed in milliseconds (ms). The methodology developed by Pons et al. [14] was applied to generate 32 candidate features: i) derivatives of time series were considered from order 0 to 10; and ii) means (denoted $m_0,...,m_{10}$) and standard deviations (denoted $sd_0,...,sd_{10}$) were computed for each time series. The following time domain measures were then computed: (1) the standard deviation of the averages of 5-s RR intervals (SDANN); (2) the mean of all the standard deviations of 5-second RR intervals (SDNNidx); (3) the standard deviation of successive differences (SDSD); (4) the standard deviation of all RR intervals (SDNN); (5) the root-mean-square of successive differences (RMSSD); (6) the percentage of differences between successive RR intervals greater than 50ms (pNN50); (7) the length of the interval, as determined by the difference between the first and third quantiles of the RR time series (IRRR); (8) the integral of the density of the RR interval histogram divided by its height (HRV.index); (9) the triangular interpolation of the RR interval histogram (TINN); and (10) the median of the absolute differences between adjacent RR intervals (MADRR). These classic indicators, which cover several domains of heart rate variability, are of primary interest in this field of analysis. They were computed using RHRV R package (Heart Rate Variability Analysis of ECG Data) [17].

### Feature selection with the $\gamma$-metric

Consider a set *S* of *n* observations $\{X_i\}_{i = 1,...,n}$, characterized by *p* features, where $X_{i} \in S \subset {\mathbb {R}}^{p}$ for $i = 1,...,n$. Let *S* be divided into *K* classes such that we have an integer vector ${\varvec{Y}}$ where $Y_{i} = 1,...,K, \forall i = 1,...,n$. For each $k \in \{1,...,K\}$, ${\varvec{W}}_{k,p}$ is the covariance matrix of the corresponding subsample of observations belonging to class *k*:

1 $$\begin{aligned} {\varvec{W_{k,p}}} = cov\left( \left\{ X_i|Y_i = k\right\} \right) , \end{aligned}$$

where ${\varvec{W_{k,p}}}$ is a diagonalizable $p\times p$ symmetrical positive semi-definite matrix in which all eigenvalues $\{\lambda _{k,j}\}_{j=1,...,p}$ are positive ($\forall j=1,...,p$, $\lambda _{k,j}\ge 0$). Let $\{\varvec{u_{k,j}}\}_{j=1,...,p}$ be the normalized eigenvectors associated with eigenvalues $\{\lambda _{k,j}\}_{j=1,...,p}$. These eigenvectors represent the direction of the *p* axes of length $\sqrt{\lambda _{k,j}}$ in a *p*-dimensional ellipse centered in ${\varvec{\mu _{k}}}$, which is the mean vector of observations in class *k*. Each class *k* ($k \in \{1,...,K\}$) is thus represented by an ellipse in ${\mathbb {R}}^{p}$.

The $\gamma$-metric is a separability measure based on the sum of the distances between each pair of classes. This measure uses positive values in the case of class separability and negative values in the case of class overlapping. More intuitively, each class of individuals is represented by an ellipse in ${\mathbb {R}}^p$, and the $\gamma$-metric represents the sum of the distances between the centroids of each pair of ellipses minus the distances between the centroids and the borders of these two ellipses. When the distance between two centroids is less than the sum of the distances separating these centroids from their respective borders, then the value of the $\gamma$-metric becomes negative. Let there be $k_{1}, k_{2} \in \{1,...,K\}$ such that $k_{1}<k_{2}$, then the algebraic distance $d_{k_{1},k_{2}}$ between the pair of classes $k_{1} \ne k_{2}$ along the mean-mean axis given by ${\varvec{\mu _{k_{1}}}\mu _{k_{2}}} = {\varvec{\mu _{k_{2}}}} - \varvec{\mu _{k_{1}}}$ can be defined as follows:

2 $$\begin{aligned} d_{k_{1},k_{2}} = \frac{1}{\alpha _{k_{1},k_{2}}}(\left\| {\varvec{\mu _{k_{1}}}\mu _{k_{2}}} \right\| -(d_{k_{1},k_{1}k_{2}}+d_{k_{2},k_{1}k_{2}})), \end{aligned}$$

where $\alpha _{k_{1},k_{2}}$ is a normalization factor defined as:

3 $$\begin{aligned} \alpha _{k_{1},k_{2}} = \sqrt{\sum _{j = 1}^{p}\lambda _{k_{1},j}} + \sqrt{\sum _{j = 1}^{p}\lambda _{k_{2},j}}, \end{aligned}$$

and $d_{k_{1},k_{1}k_{2}}$ and $d_{k_{2},k_{1}k_{2}}$ are defined as:

4 $$\begin{aligned} d_{k_{1},k_{1}k_{2}} = \frac{1}{\sqrt{\sum _{j = 1}^{p} \frac{\tilde{\mu }_{k_{1},j}^{2}}{\lambda _{k_{1},j}}}} \quad \text {and} \quad d_{k_{2},k_{1}k_{2}} = \frac{1}{\sqrt{\sum _{j = 1}^{p} \frac{\tilde{\mu }_{k_{2},j}^{2}}{\lambda _{k_{2},j}}}} , \end{aligned}$$

where $\tilde{\mu }_{k_{1},j}^{2}$ (respectively $\tilde{\mu }_{k_{2},j}^{2}$) represents the coordinates of the normalized vector ${\varvec{\mu _{k_{1}}\mu _{k_{2}}}}$ expressed in the orthogonal basis formed by the eigenvectors of ellipse $k_{1}$ (respectively $k_{2}$). If ${\varvec{U_{k_1}}}$ (respectively ${\varvec{U_{k_2}}}$) is the matrix whose columns correspond to the eigenvectors of ellispoid $k_1$ (respectively $k_2$), then the normalized mean-mean vector ${\varvec{\tilde{\mu }_{k_1}}}$ (respectively ${\varvec{\tilde{\mu }_{k_2}}}$) can be written as:

5 $$\begin{aligned} {\varvec{\tilde{\mu }_{k_1}}} = {\varvec{U_{k_1}}}^{-1} \frac{\varvec{\mu _{k_1}\mu _{k_2}}}{||{\varvec{\mu _{k_1}\mu _{k_2}}}||_2} \quad \text {and} \quad {\varvec{\tilde{\mu }_{k_2}}} = {\varvec{U_{k_2}}}^{-1} \frac{{\varvec{\mu _{k_1}\mu _{k_2}}}}{||{\varvec{\mu _{k_1}\mu _{k_2}}}||_2}. \end{aligned}$$

In other words, $d_{k_1,k_1k_2}$ represents the distance between $\mu _{k_1}$ and the border of the ellipse, and any point on this border is determined by drawing a segment between $\mu _{k_1}$ and the border of the ellipse in the same direction as ${\varvec{\mu _{k_1}\mu _{k_2}}}$. Similarly, $d_{k_2,k_1k_2}$ is the distance between $\mu _{k_2}$ and the border of the ellipse, and any point on this border is determined by drawing a segment between $\mu _{k_2}$ and the border of the ellipse in the same direction as vector $-{\varvec{\mu _{k_1}\mu _{k_2}}}$.

Finally, the $\gamma$-metric for a set of *K* classes of observations in $S \subset {\mathbb {R}}^{p}$ is defined as follows:

6 $$\begin{aligned} \gamma _{p} = \sum _{k_{1}=1}^{K} \sum _{k_{1}<k_{2}} d_{k_{1},k_{2}}. \end{aligned}$$

The above computations were performed using R and are available on GitHub [18] with all codes and data. Figure 2 illustrates the $\gamma$-metric in two scenarios: the first using a positive value of the $\gamma$-metric, the second using a negative value. A step-by-step mathematical derivation of Eq. 4 is provided in the Additional file 1.

### Feature selection stability assessment

Feature selection stability is defined as the ability of a feature selection algorithm to find the same subsets of features in similar datasets [19] or, more generally, in datasets drawn from the same distribution [20]. In this study, feature selection stability was assessed using the Kuncheva index (KI, [21]).

Let ${\mathbb {F}} = \{f_1,f_2,...,f_p\}$ be a set of *p* features. Feature selection consists in selecting a subset $S\subset {\mathbb {F}}$ containing the $p'\le p$ most relevant features based on the values of a given evaluation function. Let $s=\{S_1,S_2,...,S_{\omega }\}$ be a set of $\omega$ feature subsets obtained through $\omega$ runs of a feature selection algorithm performed on various datasets. Assuming that all elements in *s* are of the same size (i.e $|S_i|=p', \forall i \in \{1,...,\omega \}$), KI can be defined as:

7 $$\begin{aligned} \text {KI}(s) = \frac{2}{\omega (\omega -1)}\sum _{i=1}^{\omega -1}\sum _{j=i+1}^{\omega } \frac{|S_i \cap S_j|p-p'^2}{p'(p-p')}. \end{aligned}$$

This consistency index ranges from $-1$ to 1, with values close to 1 representing high feature selection stability and values close to $-1$ representing instability. By convention, the consistency index is null for $p' = p$. Values of $\omega$ were assessed by analyzing their effect on the computation of the stability index. The value that corresponded to the stabilization of KI values was retained.

### Support vector machine

The problem of discriminating between NSR and AF rhythms was treated as a supervised binary classification problem (the target variable was encoded ‘0’ for observations corresponding to NSR rhythms and as ‘1’ for observations corresponding to AF rhythms). Given the imbalance in our datasets (with AF times-series accounting for $5.79\%$ of the training dataset and for $26.72\%$ of the validation dataset), the support vector machine (SVM) [15] model seemed appropriate for our purpose of classifying heart rhythms. Indeed, SVM is a well-known classification model in high dimensional data analysis that does not require any assumption on the distribution of target values in the study sample [22]. In our study, the SVM classifier with linear kernel was used to measure the prediction performance of the $\gamma$-metric feature selection approach.

Briefly put, SVM is a machine learning model that can be used for both regression and classification. It works by identifying a hyperplane that distinctly classifies data points in a *p*-dimensional space (where *p* is the number of features). Although several hyperplanes can be selected, the SVM algorithm selects the one with the largest distance to the nearest training-data points of any class. This optimal hyperplane is then treated as a decision boundary, such that new data points falling on either side of this boundary are attributed to a different class. The model can be seen as an optimization problem in which the smallest distance between the hyperplane and the data points must be maximized. The distance between the hyperplane and the closest data points is called the margin, and the closest data points are called support vectors.

Given a training dataset of *n* points of the form $\{(x_1,y_1),...,(x_n,y_n)\} \subset {\mathbb {R}}^p \times \{-1,1\}$, we wish to find the maximum-margin hyperplane (or optimal separating hyperplane) that divides the group of points $x_i$, where $y_i = 1$ from the group of points $x_i$ where $y_i = -1$. The form of the hyperplane equation can be written as:

8 $$\begin{aligned} h(x) = {\varvec{\theta }}^\top {\varvec{x}} + b, \end{aligned}$$

where ${\varvec{\theta }} = (\theta _1,...,\theta _p)^\top \in {\mathbb {R}}^p$ and $b\in {\mathbb {R}}$. For a new data point ${\varvec{x}}$ the decision rule will be:

9 $$\begin{aligned} \hat{y} = \left\{ \begin{array}{ll} 1 &{} \text{ if } h({\varvec{x}}) \ge 0 \\ -1 &{} \text{ otherwise } \end{array}. \right. \end{aligned}$$

Thus, given the training dataset, we wish to find *h* as follows:

10 $$\begin{aligned} y_kh({\varvec{x_k}}) \ge 0, \ \forall k \in \{1,...,n\} \Leftrightarrow y_k({\varvec{\theta }}^\top {\varvec{x_k}}+b)\ge 0,\ \forall k \in \{1,...,n\}, \end{aligned}$$

where $M = \frac{1}{\Vert {\varvec{\theta }}\Vert }$ is the margin solution of $\min \limits _{1\le k \le n}y_k\ ({\varvec{\theta }}^\top \varvec{x_k}+b)$. The hyperplane is the solution of the following optimization problem:

11 $$\begin{aligned} \begin{array}{ll} &{} \max _{{\varvec{\theta }}, b} M, \\ s.t.\ &{} y_k({\varvec{\theta }}^\top {\varvec{x_k}} + b) \ge M, \ k = 1,...,n . \end{array} \end{aligned}$$

By setting $\Vert {\varvec{\theta }}\Vert = \frac{1}{M}$, we obtain the following formulation of the optimization problem:

12 $$\begin{aligned} \begin{array}{ll} &{} \min _{{\varvec{\theta }}, b}\frac{1}{2}\Vert {\varvec{\theta }}\Vert ^2 \\ s.t. \ &{} y_k({\varvec{\theta }}^\top {\varvec{x_k}}+b)\ge 1, k = 1,...,n . \end{array} \end{aligned}$$

### Strategy for feature selection in atrial fibrillation detection

In addition to the $\gamma$-metric, three other variable importance scores were computed for each individual feature. Two were derived from the Random Forest (RF) algorithm [23]: the first was the mean decrease in Gini index (MDG), which is obtained by replacing each split in each tree of the forest with its surrogate split; the second was the mean decrease in accuracy (MDA), which is obtained by randomly shuffling feature values in the out-of-bag data. Both the MDA and the MDG were computed using randomForest R package [24]. The third variable importance score was the AUC. This score is obtained using an intuitive approach: each individual feature is entered in a classification model (SVM in our case); a receiver operating characteristic (ROC) curve analysis is then conducted for each predictor; and the area under the ROC curve (AUC) is used as a measure of variable importance. The AUC was computed using caret R package [25].

The proposed strategy for feature selection in atrial fibrillation detection consists of four steps summarized as follows:

1. *Computation of variable importance scores* ($\gamma$-metric, MDA, MDG, AUC) for each individual feature of the training dataset using 150 bootstrap replications. Features were ranked in descending order for each variable importance score. The KI was measured using the 150 rankings obtained for each variable importance score.
2. *Feature ranking* in descending order according to the median value of each variable importance score for the 150 bootstrap replications.
3. *SVM fitting* of each feature ranking using 5-fold cross validation repeated 10 times on the training dataset. At each iteration, only the remaining feature with the highest variable importance value was included. For each variable importance score, 32 models were yielded. The first model contained only the most relevant feature according to the metric used, and the last one contained all the features.
4. *Computation of the classification performances* of each model on the testing folds of the 5-fold cross-validation repeated 10 times on the training dataset. Mean accuracy, specificity, sensitivity, and Matthews correlation coefficient (MCC) [26] with their standard deviations were considered. Performance results are reported in Table 3.

Finally, prediction performance was evaluated for each feature selection approach by computing median accuracy, specificity, sensitivity, and MCC with 1000 bootstrap replications of the independent validation dataset. For each performance indicator, interquartile ranges were computed as a measure of dispersion. Results are reported in Table 4.

**Table 1:** Descriptive statistics of the patients with ECG recordings. Continuous features (age, ECG recording duration) are expressed as means and standard deviations, and categorical features (gender) are expressed as absolute and relative frequencies

	AF (N = 11)	NSR (N = 23)	Total (N = 34)	*p*-value
Age (mean (sd))	68.64(15.98)	59.74(11.66)	62.62(13.63)	0.074
Gender (Male (%))	4(36.4)	14(60.9)	18(52.9)	0.331
ECG recording duration (mean (sd))	16.92(9.18)	22.17(2.62)	20.47(6.03)	0.243

The *p*-value corresponds to a Student’s t-test (respectively $\chi ^2$ test) used for continuous (respectively categorical) features

## Results

Demographic data and ECG recording duration for the 34 patients of the validation dataset are given in Table 1. Mean age was 62.62 years (68.64 in the AF group and 59.74 in the NSR group). Slightly more than half of the patients were male (36.4% in the AF group and 60.9% in the NSR group), and mean duration of ECG recordings was 20.47 hours (16.92 hours in the AF group and 22.17 hours in the NSR group). No significant difference was found between the AF and NSR groups.

Table 2 presents the means and standard deviations for each feature of the validation dataset for both the AF and NSR groups. It also shows the *p*-value between the two groups that corresponds to an unpaired bilateral Student’s means comparison test in case of normal distribution and to a Wilcoxon’s mean comparison test in case of non-normal distribution. The training dataset contained 50, 028 1-min RR interval time series and the validation dataset contained 41, 661. There was an imbalance in both the training and validation datasets due to a much higher proportion of NSR patients (94.2% in the training dataset and 73.3% in the validation dataset). No significant differences were observed for features $m_8$, $m_9$, and $m_{10}$ of the validation dataset, as shown in Table 2.

**Fig. 3:**
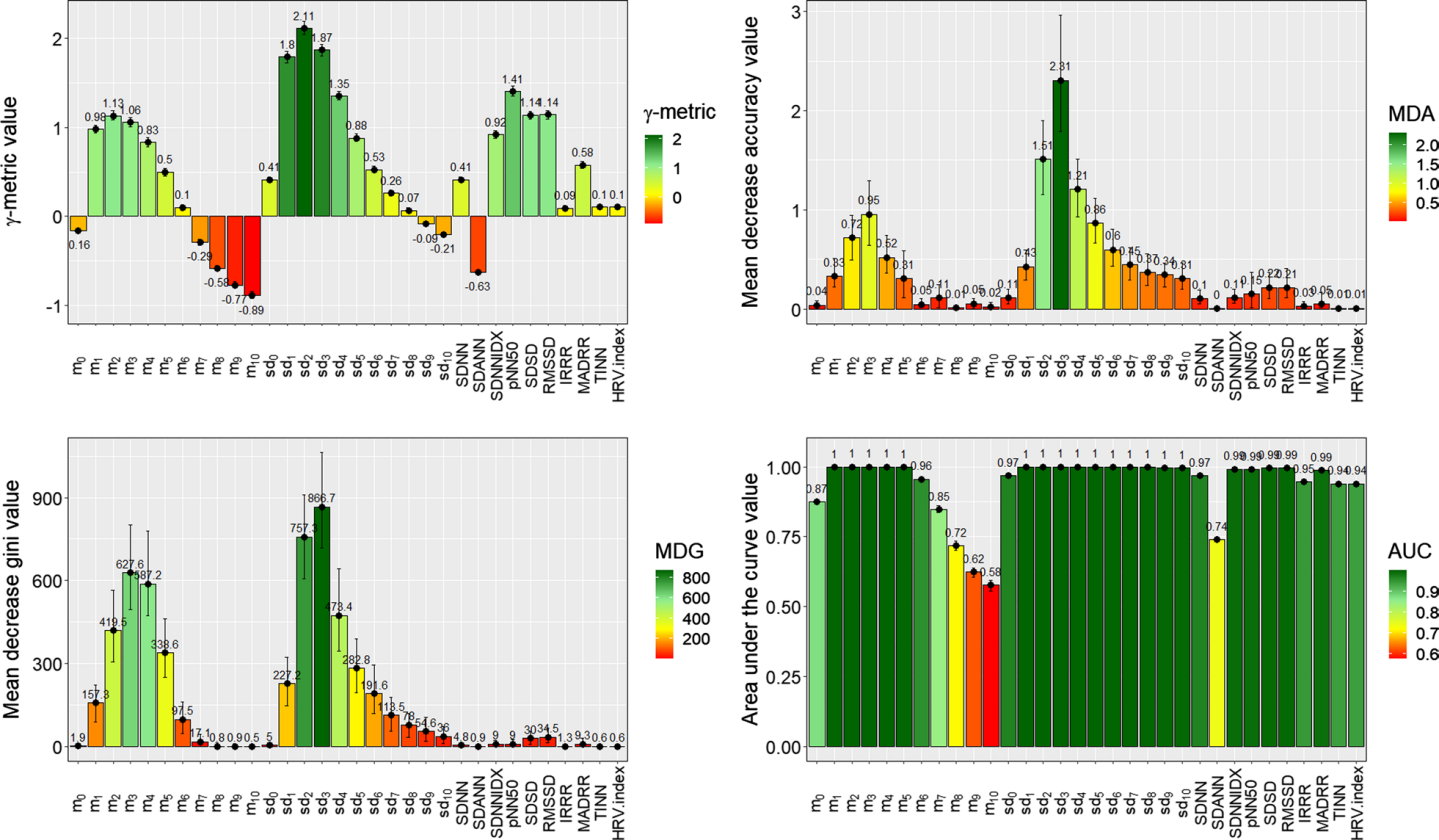
Variable importance scores of each feature obtained using the $\gamma$-metric (top-left panel), MDA (top-right panel), MDG (bottom-left panel) and AUC (bottom-right panel) feature selection approaches

Figure 3 shows the variable importance values of each feature of the training dataset obtained using the $\gamma$-metric (top-left panel), MDA (top-right panel), MDG (bottom-left panel), and AUC (bottom-right panel) approaches. When using the $\gamma$-metric approach, the three most discriminant features were $sd_{1}$, $sd_{2}$, and $sd_{3}$. This finding supports the study by Pons et al. [14], who found values that fell within the confidence intervals of our predictions, confirming the ability of the $\gamma$-metric approach to retrieve the most discriminant features. When using the MDA approach, the three most discriminant features were $sd_3$, $sd_2$, and $sd_4$. When using the MDG and AUC approaches, the three most discriminant features were $sd_3$, $sd_2$, and $m_3$.

**Fig. 4:**
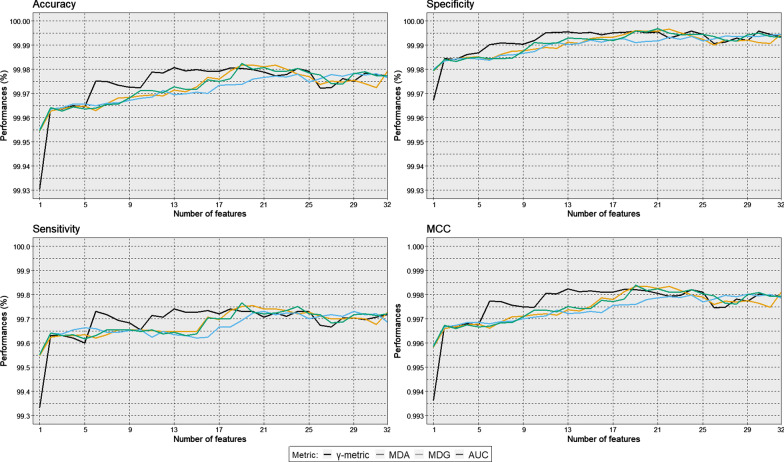
Classification performance (mean accuracy, specificity, sensitivity, and MCC values) computed on 10 replicates of 5-fold cross validation applied to the training dataset in function of the number of features and for each feature selection approach ($\gamma$-metric, MDA, MDG, and AUC)

**Table 2:** Descriptive analysis of the features of the validation dataset

Features	AF (N = 11)	NSR (N = 23)	*p*-value
$m_0$	$0.769\ (0.248)$	$0.873\ (0.114)$	0.028
$m_1$	$-0.036\ (0.016)$	$-0.002\ (0.002)$	0.000
$m_2$	$0.109\ (0.061)$	$0.006\ (0.007)$	0.000
$m_3$	$-0.273\ (0.188)$	$-0.019\ (0.023)$	0.000
$m_4$	$0.656\ (0.536)$	$0.065\ (0.085)$	0.000
$m_5$	$-1.633\ (1.508)$	$-0.252\ (0.356)$	0.000
$m_6$	$4.331\ (4.453)$	$1.104\ (1.602)$	0.002
$m_7$	$-13.642\ (16.670)$	$-5.073\ (7.710)$	0.015
$m_8$	$53.254\ (91.939)$	$24.687\ (38.695)$	0.071
$m_9$	$-288.073\ (666.902)$	$-124.233\ (203.657)$	0.243
$m_{10}$	$1901.032 \ (5304.157)$	$648.314\ (1122.787)$	0.690
$sd_0$	$0.142\ (0.037)$	$0.047\ (0.019)$	0.000
$sd_1$	$0.270\ (0.069)$	$0.042\ (0.020)$	0.000
$sd_2$	$0.714\ (0.296)$	$0.097\ (0.057)$	0.000
$sd_3$	$2.163\ (1.235)$	$0.297\ (0.240)$	0.000
$sd_4$	$7.190\ (5.080)$	$1.077\ (1.071)$	0.000
$sd_5$	$25.866\ (21.270)$	$4.355\ (4.916)$	0.000
$sd_6$	$99.809\ (91.928)$	$18.842\ (23.025)$	0.001
$sd_7$	$411.791\ (416.661)$	$85.683\ (111.063)$	0.005
$sd_8$	$1822.127\ (2043.844)$	$404.282\ (549.422)$	0.008
$sd_9$	$8773.333\ (11{,}389.615)$	$1976.371\ (2815.619)$	0.026
$sd_{10}$	$46{,}563.114\ (73{,}918.526)$	$9916.219\ (14{,}751.188)$	0.042
SDNN	$141.717\ (37.248)$	$46.580\ (18.907)$	0.000
SDANN	$60.200\ (24.675)$	$35.914\ (14.735)$	0.010
SDNNIDX	$131.858\ (32.635)$	$25.081\ (12.269)$	0.000
pNN50	$74.878\ (6.375)$	$10.601\ (11.328)$	0.000
SDSD	$195.287\ (47.089)$	$35.023\ (18.044)$	0.000
RMSSD	$193.983\ (46.599)$	$34.779\ (17.902)$	0.000
IRRR	$185.401\ (54.637)$	$57.067\ (26.341)$	0.000
MADRR	$123.929\ (30.981)$	$17.391\ (11.339)$	0.000
TINN	$247.492\ (27.711)$	$114.821\ (33.433)$	0.000
HRV.index	$15.839\ (1.773)$	$7.349\ (2.140)$	0.000

For each patient, we computed the mean value of the features derived from each of his or her 1-min RR interval. Each line corresponds to the mean values and standard deviations of all patients in the AF and NSR groups. The *p*-value corresponds to a Student’s t-test in case of normal of normal distribution and to a Mann–Whitney U test in case of non-normal distribution

The third column in Table 3 shows the stability performance of the four feature selection approaches, as measured by the KI values obtained for each model using the training dataset (the name of the feature selected at each step is specified in the second column). For a number of selected features $p < 16$ (i.e., less than half of the initial features), the KI values obtained using the $\gamma$-metric approach were higher than those obtained using RF-based (MDA and MDG) approaches. This superiority of the $\gamma$-metric approach was no longer observed for $p > 16$, with the AUC approach then yielding the highest KI values (results not shown).

Figure 4 presents the mean test accuracy, specificity, sensitivity, and MCC values for each model fitted on the 10 replicates of the 5-fold cross validation applied to the training dataset (models with one to 32 features). The $\gamma$-metric approach outperformed the three other approaches for all indicators, especially for $p' \le 16$. Maximal accuracy for the $\gamma$-metric approach (99.98%) was observed for $p' = 13$ features. Likewise, maximal MCC for the $\gamma$-metric approach (0.998) was observed for $p' = 13$. Specificity and sensitivity increased until $p'=16$, and remained constant with higher number of features for all four approaches. Table 3 presents these same values for models with one to 16 features. In most cases, the $\gamma$-metric approach outperformed the other approaches in terms of accuracy, specificity, sensitivity, and MCC. Of the two RF-based approaches, the MDA approach showed the best indicators.

**Fig. 5:**
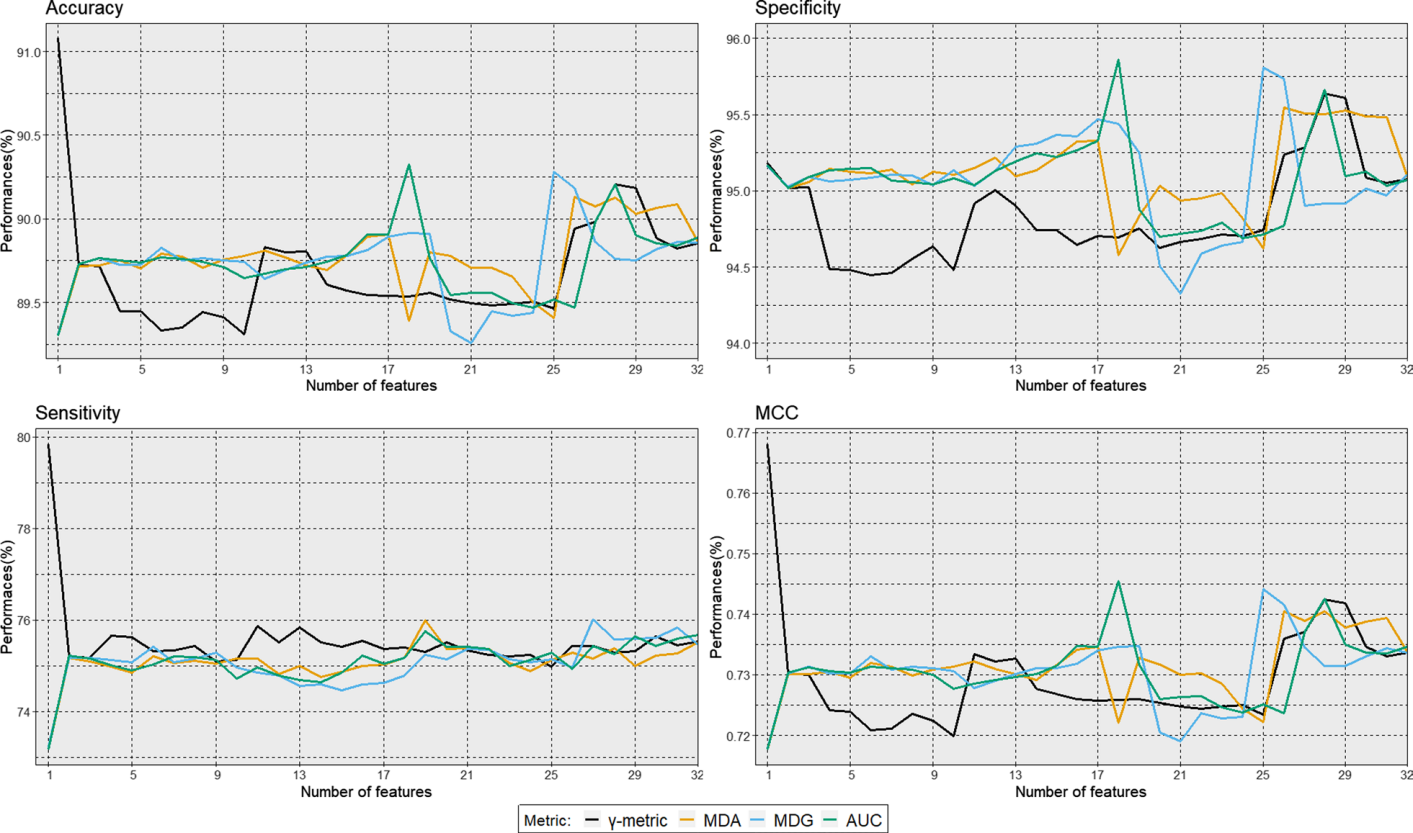
Classification performance (median accuracy, specificity, sensitivity, and MCC values) computed by bootstrap with 1000 replications applied to the validation dataset in function of the number of features and for each feature selection approach ($\gamma$-metric, MDA, MDG, and AUC). Interquartile ranges were computed over the bootstrap estimations of the performance indicators as a measure of dispersion

Figure 5 presents the median accuracy, specificity, sensitivity, and MCC values for each model calculated on boostrap samples of the validation dataset (models with one to 32 features). Table 4 presents these same values for models with one to 16 features. Surprisingly, maximal accuracy was observed for the $\gamma$-metric approach for $p'=1$ (using only feature $sd_2$). This approach outperformed all others for $p'=13$ with an accuracy of 89.8%, corroborating our earlier test set results obtained through 5-fold cross validation. It also outperformed all other approaches for the MCC indicator, with a value of 0.73 for $p'=13$. However, the $\gamma$-metric approach did not have the highest specificity; moreover, the lowest specificity was 94.45 for $p'=6$. On the other hand, sensitivity values for the $\gamma$-metric approach corroborated our earlier test set results, with the highest sensitivity observed for $p'\le 18$.

The $\gamma$-metric feature selection approach, which is a filter method, had the shortest running time at 0.31 s. The other three feature selection approaches are wrapper methods, and therefore involve a learning phase. The MDG and MDA approaches were applied simultaneously and had the longest running time at 65.17 s. The AUC approach, which was applied using an SVM classification model, had a running time of 3.09 s. Average running times were computed using 10 bootstrap samples from the training dataset with Intel(R) Xeon(R) W-2104 CPU at 3.20GHz, on a 64-bits system.

**Table 3:** Classification performances (mean accuracy, specificity, sensitivity, and MCC values with their standard deviations) computed on 10 replicates of 5-fold cross validation applied to the training dataset

Feature selection (Training dataset)			Classification performances (Training dataset)			
Dim	Added feature	KI	Accuracy	Specificity	Sensitivity	MCC
$\gamma$*-metric*						
1	$sd_2$	1.000	$0.9993\ (0.000228)$	$0.9997\ (0.000172)$	$0.9933\ (0.000030)$	$0.994\ (0.0021)$
2	$sd_3$	1.000	$0.9996\ (0.000164)$	$0.9997\ (0.000127)$	$0.9963\ (0.000024)$	$0.997\ (0.0015)$
3	$sd_1$	1.000	$0.9996\ (0.000181)$	$0.9998\ (0.000134)$	$0.9963\ (0.000025)$	$0.997\ (0.0017)$
4	pNN50	0.971	$0.9997\ (0.000174)$	$0.9999\ (0.000139)$	$0.9962\ (0.000019)$	$0.997\ (0.0016)$
5	$sd_4$	1.000	$0.9996\ (0.000197)$	$0.9999\ (0.000104)$	$0.9960\ (0.000026)$	$0.997\ (0.0018)$
6	RMSSD	0.926	$0.9998\ (0.000133)$	$0.9999\ (0.000080)$	$0.9973\ (0.000018)$	$0.998\ (0.0012)$
7	SDSD	0.918	$0.9998\ (0.000143)$	$0.9999\ (0.000093)$	$0.9972\ (0.000017)$	$0.998\ (0.0013)$
8	$m_2$	1.000	$0.9997\ (0.000145)$	$0.9999\ (0.000085)$	$0.9969\ (0.000023)$	$0.998\ (0.0013)$
9	$m_3$	1.000	$0.9997\ (0.000145)$	$0.9999\ (0.000086)$	$0.9968\ (0.000021)$	$0.997\ (0.0013)$
10	$m_1$	1.000	$0.9997\ (0.000151)$	$0.9999\ (0.000100)$	$0.9966\ (0.000026)$	$0.997\ (0.0014)$
11	SDNNIDX	0.981	$0.9998\ (0.000132)$	$1.0000\ (0.000065)$	$0.9971\ (0.000021)$	$0.998\ (0.0012)$
12	$sd_5$	0.995	$0.9998\ (0.000152)$	$1.0000\ (0.000065)$	$0.9971\ (0.000024)$	$0.998\ (0.0014)$
13	$m_4$	1.000	$0.9998\ (0.000154)$	$1.0000\ (0.000061)$	$0.9974\ (0.000022)$	$0.998\ (0.0014)$
14	MADRR	0.990	$0.9998\ (0.000136)$	$0.9999\ (0.000072)$	$0.9973\ (0.000021)$	$0.998\ (0.0012)$
15	$sd_6$	0.967	$0.9998\ (0.000132)$	$1.0000\ (0.000065)$	$0.9973\ (0.000021)$	$0.998\ (0.0012)$
16	$m_5$	1.000	$0.9998\ (0.000134)$	$0.9999\ (0.000091)$	$0.9973\ (0.000018)$	$0.998\ (0.0012)$
*Mean decrease accuracy*						
1	$sd_3$	1.000	$0.9995\ (0.000209)$	$0.9998\ (0.000151)$	$0.9955\ (0.002087)$	$0.996\ (0.0019)$
2	$sd_2$	0.876	$0.9996\ (0.000162)$	$0.9998\ (0.000136)$	$0.9962\ (0.002022)$	$0.996\ (0.0015)$
3	$sd_4$	0.902	$0.9996\ (0.000175)$	$0.9998\ (0.000127)$	$0.9963\ (0.002363)$	$0.997\ (0.0016)$
4	$m_3$	0.852	$0.9996\ (0.000220)$	$0.9998\ (0.000144)$	$0.9963\ (0.003057)$	$0.997\ (0.0020)$
5	$sd_5$	0.868	$0.9996\ (0.000179)$	$0.9999\ (0.000130)$	$0.9963\ (0.002221)$	$0.997\ (0.0016)$
6	$m_2$	0.886	$0.9996\ (0.000151)$	$0.9998\ (0.000114)$	$0.9962\ (0.002100)$	$0.997\ (0.0014)$
7	$sd_6$	0.883	$0.9997\ (0.000171)$	$0.9999\ (0.000114)$	$0.9963\ (0.002735)$	$0.997\ (0.0016)$
8	$m_4$	0.858	$0.9997\ (0.000122)$	$0.9999\ (0.000100)$	$0.9966\ (0.001843)$	$0.997\ (0.0011)$
9	$sd_7$	0.838	$0.9997\ (0.000161)$	$0.9999\ (0.000094)$	$0.9966\ (0.002583)$	$0.997\ (0.0015)$
10	$sd_1$	0.815	$0.9997\ (0.000177)$	$0.9999\ (0.000120)$	$0.9966\ (0.002512)$	$0.997\ (0.0016)$
11	$sd_8$	0.802	$0.9997\ (0.000171)$	$0.9999\ (0.000109)$	$0.9965\ (0.002426)$	$0.997\ (0.0016)$
12	$sd_9$	0.796	$0.9997\ (0.000161)$	$0.9999\ (0.000081)$	$0.9965\ (0.002487)$	$0.997\ (0.0015)$
13	$m_1$	0.801	$0.9997\ (0.000156)$	$0.9999\ (0.000119)$	$0.9965\ (0.002089)$	$0.997\ (0.0014)$
14	$sd_{10}$	0.824	$0.9997\ (0.000191)$	$0.9999\ (0.000102)$	$0.9965\ (0.002697)$	$0.997\ (0.0018)$
15	$m_5$	0.851	$0.9997\ (0.000144)$	$0.9999\ (0.000090)$	$0.9965\ (0.002118)$	$0.998\ (0.0013)$
16	SDSD	0.853	$0.9998\ (0.000151)$	$0.9999\ (0.000070)$	$0.9971\ (0.002154)$	$0.998\ (0.0014)$
*Mean decrease gini*						
1	$sd_3$	0.634	$0.9996\ (0.000210)$	$0.9998\ (0.000128)$	$0.9955\ (0.002808)$	$0.996\ (0.0019)$
2	$sd_2$	0.778	$0.9996\ (0.000176)$	$0.9998\ (0.000112)$	$0.9964\ (0.002226)$	$0.997\ (0.0016)$
3	$m_3$	0.790	$0.9996\ (0.000157)$	$0.9998\ (0.000101)$	$0.9964\ (0.002343)$	$0.997\ (0.0014)$
4	$m_4$	0.883	$0.9997\ (0.000154)$	$0.9998\ (0.000118)$	$0.9966\ (0.002061)$	$0.997\ (0.0014)$
5	$sd_4$	0.886	$0.9997\ (0.000164)$	$0.9998\ (0.000147)$	$0.9966\ (0.001517)$	$0.997\ (0.0015)$
6	$m_2$	0.911	$0.9997\ (0.000173)$	$0.9998\ (0.000136)$	$0.9966\ (0.002243)$	$0.997\ (0.0016)$
7	$m_5$	0.910	$0.9997\ (0.000206)$	$0.9999\ (0.000113)$	$0.9964\ (0.002639)$	$0.997\ (0.0019)$
8	$sd_5$	0.915	$0.9997\ (0.000135)$	$0.9999\ (0.000106)$	$0.9964\ (0.002013)$	$0.997\ (0.0012)$
9	$sd_1$	0.914	$0.9997\ (0.000168)$	$0.9999\ (0.000113)$	$0.9965\ (0.002046)$	$0.997\ (0.0015)$
10	$sd_6$	0.929	$0.9997\ (0.000162)$	$0.9999\ (0.000115)$	$0.9966\ (0.002147)$	$0.997\ (0.0015)$
11	$m_1$	0.931	$0.9997\ (0.000176)$	$0.9999\ (0.000109)$	$0.9962\ (0.002669)$	$0.997\ (0.0016)$
12	$sd_7$	0.912	$0.9997\ (0.000151)$	$0.9999\ (0.000108)$	$0.9965\ (0.002256)$	$0.997\ (0.0014)$
13	$m_6$	0.919	$0.9997\ (0.000128)$	$0.9999\ (0.000091)$	$0.9963\ (0.001798)$	$0.997\ (0.0012)$
14	$sd_8$	0.922	$0.9997\ (0.000170)$	$0.9999\ (0.000085)$	$0.9963\ (0.002337)$	$0.997\ (0.0016)$
15	$sd_9$	0.912	$0.9997\ (0.000156)$	$0.9999\ (0.000082)$	$0.9962\ (0.002653)$	$0.997\ (0.0014)$
16	$sd_{10}$	0.880	$0.9997\ (0.000143)$	$0.9999\ (0.000102)$	$0.9962\ (0.002302)$	$0.997\ (0.0013)$
*AUC*						
1	$sd_3$	1.000	$0.9995\ (0.000193)$	$0.9998\ (0.000149)$	$0.9955\ (0.002061)$	$0.996\ (0.0018)$
2	$sd_2$	0.861	$0.9996\ (0.000163)$	$0.9998\ (0.000114)$	$0.9964\ (0.002626)$	$0.997\ (0.0015)$
3	$m_3$	0.802	$0.9996\ (0.000182)$	$0.9998\ (0.000132)$	$0.9963\ (0.002337)$	$0.997\ (0.0017)$
4	$sd_4$	0.857	$0.9996\ (0.000184)$	$0.9998\ (0.000122)$	$0.9963\ (0.002353)$	$0.997\ (0.0017)$
5	$m_2$	0.879	$0.9996\ (0.000191)$	$0.9998\ (0.000109)$	$0.9962\ (0.002470)$	$0.997\ (0.0018)$
6	$sd_5$	0.897	$0.9996\ (0.000165)$	$0.9998\ (0.000110)$	$0.9963\ (0.002512)$	$0.997\ (0.0015)$
7	$m_4$	0.924	$0.9997\ (0.000150)$	$0.9998\ (0.000129)$	$0.9966\ (0.002119)$	$0.997\ (0.0014)$
8	$sd_1$	0.970	$0.9997\ (0.000172)$	$0.9998\ (0.000136)$	$0.9966\ (0.002388)$	$0.997\ (0.0016)$
9	$sd_6$	0.978	$0.9997\ (0.000115)$	$0.9999\ (0.000093)$	$0.9966\ (0.002001)$	$0.997\ (0.0011)$
10	$m_1$	0.907	$0.9997\ (0.000186)$	$0.9999\ (0.000097)$	$0.9965\ (0.002535)$	$0.997\ (0.0017)$
11	$sd_7$	0.924	$0.9997\ (0.000149)$	$0.9999\ (0.000087)$	$0.9966\ (0.001939)$	$0.997\ (0.0014)$
12	$m_5$	0.941	$0.9997\ (0.000154)$	$0.9999\ (0.000098)$	$0.9964\ (0.002420)$	$0.997\ (0.0014)$
13	$sd_8$	0.995	$0.9997\ (0.000138)$	$0.9999\ (0.000082)$	$0.9964\ (0.002398)$	$0.998\ (0.0013)$
14	$sd_9$	1.000	$0.9997\ (0.000135)$	$0.9999\ (0.000087)$	$0.9964\ (0.001707)$	$0.997\ (0.0012)$
15	$sd_{10}$	0.947	$0.9997\ (0.000159)$	$0.9999\ (0.000094)$	$0.9964\ (0.002237)$	$0.997\ (0.0015)$
16	RMSSD	0.951	$0.9998\ (0.000162)$	$0.9999\ (0.000094)$	$0.9970\ (0.002176)$	$0.998\ (0.0015)$

Results are presented according to the number of features (from 1 to 16) and to the KI calculated on 150 replications of the ranking given by the four feature selection approaches ($\gamma$-metric, MDA, MDG, and AUC)

**Table 4:** Classification performances (median accuracy, sensitivity, specificity, and MCC values with their interquartile ranges) computed by bootstrap with 1000 replications applied to the validation dataset

Features		Classification performances (Validation dataset)			
Dim	Added feature	Accuracy	Specificity	Sensitivity	MCC
$\gamma$*-metric*					
1	$sd_2$	$0.9108\ (0.0019)$	$0.9518\ (0.0016)$	$0.7983\ (0.0054)$	$0.768\ (0.0051)$
2	$sd_3$	$0.8973\ (0.0020)$	$0.9502\ (0.0017)$	$0.7520\ (0.0051)$	$0.730\ (0.0053)$
3	$sd_1$	$0.8972\ (0.0019)$	$0.9502\ (0.0017)$	$0.7518\ (0.0054)$	$0.730\ (0.0052)$
4	pNN50	$0.8945\ (0.0022)$	$0.9449\ (0.0018)$	$0.7565\ (0.0055)$	$0.724\ (0.0054)$
5	$sd_4$	$0.8945\ (0.0020)$	$0.9448\ (0.0017)$	$0.7563\ (0.0054)$	$0.724\ (0.0051)$
6	RMSSD	$0.8933\ (0.0020)$	$0.9445\ (0.0018)$	$0.7531\ (0.0051)$	$0.721\ (0.0052)$
7	SDSD	$0.8935\ (0.0021)$	$0.9446\ (0.0018)$	$0.7533\ (0.0058)$	$0.721\ (0.0054)$
8	$m_2$	$0.8944\ (0.0021)$	$0.9456\ (0.0018)$	$0.7543\ (0.0057)$	$0.724\ (0.0053)$
9	$m_3$	$0.8941\ (0.0020)$	$0.9464\ (0.0018)$	$0.7509\ (0.0057)$	$0.722\ (0.0052)$
10	$m_1$	$0.8931\ (0.0021)$	$0.9448\ (0.0018)$	$0.7512\ (0.0056)$	$0.720\ (0.0052)$
11	SDNNIDX	$0.8983\ (0.0021)$	$0.9492\ (0.0016)$	$0.7586\ (0.0056)$	$0.733\ (0.0054)$
12	$sd_5$	$0.8980\ (0.0021)$	$0.9500\ (0.0017)$	$0.7552\ (0.0057)$	$0.732\ (0.0052)$
13	$m_4$	$0.8980\ (0.0019)$	$0.9490\ (0.0016)$	$0.7583\ (0.0058)$	$0.733\ (0.0050)$
14	MADDR	$0.8961\ (0.0020)$	$0.9474\ (0.0016)$	$0.7551\ (0.0056)$	$0.728\ (0.0054)$
15	$sd_6$	$0.8957\ (0.0021)$	$0.9474\ (0.0018)$	$0.7542\ (0.0052)$	$0.727\ (0.0054)$
16	$m_5$	$0.8955\ (0.0021)$	$0.9465\ (0.0018)$	$0.7554\ (0.0055)$	$0.726\ (0.0054)$
*Mean decrease accuracy*					
1	$sd_3$	$0.8930\ (0.0022)$	$0.9517\ (0.0017)$	$0.7317\ (0.0059)$	$0.718\ (0.0060)$
2	$sd_2$	$0.8972\ (0.0021)$	$0.9502\ (0.0017)$	$0.7517\ (0.0051)$	$0.730\ (0.0055)$
3	$sd_4$	$0.8972\ (0.0019)$	$0.9506\ (0.0017)$	$0.7509\ (0.0053)$	$0.730\ (0.0053)$
4	$m_3$	$0.8975\ (0.0020)$	$0.9514\ (0.0014)$	$0.7498\ (0.0055)$	$0.731\ (0.0051)$
5	$sd_5$	$0.8970\ (0.0020)$	$0.9513\ (0.0017)$	$0.7484\ (0.0054)$	$0.729\ (0.0053)$
6	$m_2$	$0.8979\ (0.0019)$	$0.9512\ (0.0017)$	$0.7520\ (0.0052)$	$0.732\ (0.0051)$
7	$sd_6$	$0.8977\ (0.0020)$	$0.9514\ (0.0016)$	$0.7506\ (0.0059)$	$0.731\ (0.0052)$
8	$m_4$	$0.8971\ (0.0021)$	$0.9504\ (0.0017)$	$0.7510\ (0.0057)$	$0.730\ (0.0053)$
9	$sd_7$	$0.8976\ (0.0019)$	$0.9512\ (0.0017)$	$0.7505\ (0.0057)$	$0.731\ (0.0051)$
10	$sd_1$	$0.8978\ (0.0020)$	$0.9511\ (0.0016)$	$0.7516\ (0.0059)$	$0.731\ (0.0051)$
11	$sd_8$	$0.8981\ (0.0021)$	$0.9515\ (0.0016)$	$0.7516\ (0.0060)$	$0.732\ (0.0055)$
12	$sd_9$	$0.8977\ (0.0021)$	$0.9522\ (0.0016)$	$0.7482\ (0.0057)$	$0.731\ (0.0053)$
13	$m_1$	$0.8972\ (0.0020)$	$0.9510\ (0.0016)$	$0.7499\ (0.0058)$	$0.730\ (0.0052)$
14	$sd_{10}$	$0.8970\ (0.0020)$	$0.9514\ (0.0016)$	$0.7475\ (0.0055)$	$0.729\ (0.0054)$
15	$m_5$	$0.8978\ (0.0021)$	$0.9522\ (0.0016)$	$0.7487\ (0.0055)$	$0.732\ (0.0054)$
16	SDSD	$0.8989\ (0.0020)$	$0.9532\ (0.0017)$	$0.7499\ (0.0054)$	$0.734\ (0.0053)$
*Mean decrease gini*					
1	$sd_3$	$0.8931\ (0.0022)$	$0.9516\ (0.0017)$	$0.7321\ (0.0058)$	$0.718\ (0.0056)$
2	$sd_2$	$0.8973\ (0.0021)$	$0.9503\ (0.0017)$	$0.7517\ (0.0051)$	$0.730\ (0.0054)$
3	$m_3$	$0.8977\ (0.0019)$	$0.9509\ (0.0016)$	$0.7517\ (0.0054)$	$0.731\ (0.0051)$
4	$m_4$	$0.8972\ (0.0021)$	$0.9506\ (0.0017)$	$0.7512\ (0.0056)$	$0.730\ (0.0053)$
5	$sd_4$	$0.8973\ (0.0019)$	$0.9507\ (0.0016)$	$0.7507\ (0.0052)$	$0.730\ (0.0051)$
6	$m_2$	$0.8983\ (0.0019)$	$0.9509\ (0.0016)$	$0.7541\ (0.0050)$	$0.733\ (0.0051)$
7	$m_5$	$0.8975\ (0.0020)$	$0.9510\ (0.0017)$	$0.7507\ (0.0060)$	$0.731\ (0.0052)$
8	$sd_5$	$0.8977\ (0.0020)$	$0.9510\ (0.0017)$	$0.7515\ (0.0057)$	$0.731\ (0.0053)$
9	$sd_1$	$0.8975\ (0.0020)$	$0.9504\ (0.0017)$	$0.7528\ (0.0056)$	$0.731\ (0.0051)$
10	$sd_6$	$0.8974\ (0.0020)$	$0.9513\ (0.0016)$	$0.7497\ (0.0059)$	$0.731\ (0.0052)$
11	$m_1$	$0.8964\ (0.0020)$	$0.9504\ (0.0016)$	$0.7486\ (0.0060)$	$0.728\ (0.0055)$
12	$sd_7$	$0.8970\ (0.0021)$	$0.9513\ (0.0017)$	$0.7478\ (0.0060)$	$0.729\ (0.0054)$
13	$m_6$	$0.8974\ (0.0020)$	$0.9529\ (0.0015)$	$0.7456\ (0.0060)$	$0.730\ (0.0052)$
14	$sd_8$	$0.8977\ (0.0021)$	$0.9531\ (0.0015)$	$0.7459\ (0.0056)$	$0.731\ (0.0053)$
15	$sd_9$	$0.8978\ (0.0021)$	$0.9537\ (0.0016)$	$0.7447\ (0.0056)$	$0.731\ (0.0055)$
16	$sd_{10}$	$0.8981\ (0.0020)$	$0.9536\ (0.0017)$	$0.7459\ (0.0055)$	$0.732\ (0.0052)$
*AUC*					
1	$sd_3$	$0.8930\ (0.0022)$	$0.9517\ (0.0017)$	$0.7317\ (0.0059)$	$0.718\ (0.0056)$
2	$sd_2$	$0.8973\ (0.0020)$	$0.9502\ (0.0017)$	$0.7317\ (0.0059)$	$0.730\ (0.0053)$
3	$m_3$	$0.8977\ (0.0019)$	$0.9509\ (0.0016)$	$0.7516\ (0.0055)$	$0.731\ (0.0051)$
4	$sd_4$	$0.8975\ (0.0020)$	$0.9514\ (0.0017)$	$0.7501\ (0.0056)$	$0.731\ (0.0053)$
5	$m_2$	$0.8974\ (0.0020)$	$0.9515\ (0.0016)$	$0.7490\ (0.0054)$	$0.730\ (0.0052)$
6	$sd_5$	$0.8977\ (0.0019)$	$0.9515\ (0.0017)$	$0.7502\ (0.0051)$	$0.731\ (0.0052)$
7	$m_4$	$0.8976\ (0.0020)$	$0.9507\ (0.0016)$	$0.7520\ (0.0059)$	$0.731\ (0.0052)$
8	$sd_1$	$0.8974\ (0.0021)$	$0.9506\ (0.0017)$	$0.7518\ (0.0056)$	$0.731\ (0.0053)$
9	$sd_6$	$0.8971\ (0.0019)$	$0.9504\ (0.0017)$	$0.7512\ (0.0057)$	$0.730\ (0.0052)$
10	$m_1$	$0.8965\ (0.0020)$	$0.9508\ (0.0016)$	$0.7473\ (0.0060)$	$0.728\ (0.0051)$
11	$sd_7$	$0.8967\ (0.0021)$	$0.9504\ (0.0016)$	$0.7496\ (0.0059)$	$0.729\ (0.0054)$
12	$m_5$	$0.8970\ (0.0021)$	$0.9513\ (0.0017)$	$0.7478\ (0.0060)$	$0.729\ (0.0054)$
13	$sd_8$	$0.8971\ (0.0020)$	$0.9519\ (0.0015)$	$0.7470\ (0.0057)$	$0.730\ (0.0052)$
14	$sd_9$	$0.8974\ (0.0020)$	$0.9525\ (0.0016)$	$0.7464\ (0.0055)$	$0.730\ (0.0054)$
15	$sd_{10}$	$0.8978\ (0.0021)$	$0.9522\ (0.0016)$	$0.7486\ (0.0055)$	$0.731\ (0.0054)$
16	RMSSD	$0.8991\ (0.0020)$	$0.9526\ (0.0018)$	$0.7522\ (0.0055)$	$0.735\ (0.0051)$

Results are presented according to the number of features (from 1 to 16) selected by each feature selection approach ($\gamma$-metric, MDA, MDG, and AUC). Interquartile ranges were computed on the bootstrap estimations of the performance indicators as a measure of dispersion

## Discussion

In this paper, we described a filter approach for feature selection in classification using an evaluation function, the $\gamma$-metric, which assesses separability between classes. We compared this feature selection approach to state-of-the-art approaches using two independent datasets containing 1-min RR interval time series of heart rhythms (NSR and AF) in a context of supervised binary classification.

This $\gamma$-metric feature selection approach showed satisfactory results and outperformed almost all other approaches both in terms of classification performance (which was computed using test sets obtained through cross-validation applied to the training dataset as well as validation sets obtained through bootstrap resampling applied to the validation dataset) and feature selection stability (which was computed using bootstrap resampling applied to the training dataset). While there was an imbalance in our two datasets (as the prevalence of NSR is much higher than that of AF), this problem was accounted for by using the SVM model as a classifier and the MCC [26] as a classification performance index.

The main advantage of the proposed approach is its filtering process. Unlike wrapper methods [10], which evaluate the selected features using the performance of a given classifier, filter methods are model-agnostic and select features without employing a classifier, which is less greedy in terms of running time.

This study also examined the stability of the feature selection performed by the $\gamma$-metric approach. In the field of classification, very large and very small sample sizes have presented a major challenge for researchers in recent years because they can lead to instability. Assessing the stability of our feature selection algorithm, and not just its classification performance, seemed to us important in this context. A taxonomy of feature selection stability indices has already been proposed in the literature [27]. While other indices such as the weighted consistency index and the average Tanimoto index are available for more general cases (for instance, when the number of selected features is variable), we chose to use the KI because it implies that the feature subsets obtained with different approaches all have the same size.

With the development of connected medical devices, automatic AF detection has become a source of hope, as it can be used to detect the onset of fibrillation early on and therefore to provide appropriate medical care. As our study indicates, whatever the variable importance score computed for each individual feature ($\gamma$-metric, MDA, MDG, AUC), $sd_2$ and $sd_3$ are the most discriminant of the 32 candidate features covering different domains of heart rate variability. As such, they are of primary interest for heart rhythm characterization. The classification performance of the $\gamma$-metric feature selection approach was found to be stable even with only five features ($sd_2$, $sd_3$, $sd_1$, pNN50, and $sd_4$). Not only do our results support those of Pons et al. [14], but they confirm the advantages of the $\gamma$-metric approach for a wider range of features. Moreover, our algorithm performed well despite the imbalance in our datasets, an imbalance that reflects conditions in the real world where NSR is more prevalent than AF.

The $\gamma$-metric feature selection approach was found to be very efficient for heart rhythm characterization using ECG recordings. However, extrapolation to other fields of application should only be made with caution. While Pons et al. thoroughly examined the robustness of the $\gamma$-metric approach using perturbated data and induced RR interval time series [14], we compared the approach to other feature selection tools using more clear-cut data (AF versus NSR). This means that our algorithm was presented with a relatively easy classification problem, as shown by the high accuracy values. Accordingly, we should not expect to obtain similar results in more complex classification tasks, for instance when dealing with unusual heart rhythms and ECG dynamics (high density of polymorphic ectopic beats, slow AF, conduction disorders, large R waves, etc.) or with more heterogeneous datasets as found in oncology.

In our study, the SVM model was used as a classifier [15]. No other classifier was considered because our aim was to compare feature selection approaches in terms of classification performance, and stability, not to compare classifiers. By contrast, in their study, Pons et al. [14] used an unregularized logistic regression classifier trained on a dataset containing 22 features. Our approach could be improved by using other benchmark classifiers, particularly those that are employed in machine learning tasks: classification and regression trees (CART [28]) and their RF extensions [23] or gradient boosting machine [29]. These supervised learning approaches can serve as classification methods for this kind of study; they also provide variable importance scores that can be used for feature selection.

There was an imbalance in our datasets, as the percentage of NSR patients was much higher in both datasets (94.2% in the training dataset and 73.3% in the validation dataset). While the SVM model works fine on sparse and imbalanced data, and while accuracy values obtained for the two datasets were significantly greater than the percentage of observations in the most occurring class (NSR), this imbalance may have biased our estimates [30]. One solution to the problem of imbalance may be to assign different weights to individuals depending on the class they belong to, with weights attributing a greater misclassification penalty to individuals in the least occurring class (AF). Another solution may be to over-sample observations in the least occurring class, or, conversely, to under-sample observations in the most occurring class. Lastly, one could use the Synthetic Minority Over-sampling Technique (SMOTE) [31], a method that combines over-sampling with under-sampling to improve the performance of the classifier. We did not use the weighted SVM or SMOTE in our study, as the performance results obtained with the current SVM version were satisfactory in terms of prediction and stability. As such, we were able to use the exact same model for each feature selection approach considered, which was important for comparability purposes. For similar reasons of comparability, we did not run a hyper-parameter tuning of the SVM model, nor did we use other kernels. Future studies should compare the results obtained with different versions of the SVM model using other kernels (linear, polynomial, Gaussian, radial, etc.) to better account for non-linear effects.

Similarly, multi-layer neural networks may be a good alternative to more traditional machine learning models. Indeed, these networks use non-linear activation functions (rectified linear, hyperbolic tangent, and sigmoid activation functions being the most common) that can lead to significantly better performance results. A recent literature review [32] has described different applications of deep learning models in healthcare using physiological data; it has concluded by recommending these models to improve diagnostic performance. In the field of automatic atrial detection, the use of convolutional neural networks [33] seems to yield more accurate and robust results. Another study has described the advantages of using deep neural networks in the field of AF detection when considering more than three classes (for example, NSR, AF, and noise) [34]. More recently, a method based on deep learning and feature extraction (MultiFusionNet, [35]) has been proposed that outperforms most recent algorithms. The main advantage of this method is that it combines extracted features with raw data to train the deep classifier, which can be construed as a form of feature selection. Future evaluations of the $\gamma$-metric feature selection approach should consider using such methods.

In the future, studies should be conducted to evaluate the performance of the $\gamma$-metric feature selection approach for a greater number of classes. In particular, it would be interesting to determine the impact of using a greater number of classes on the selected variables along with error rates and stability indices. In the field of automated AF detection, this approach could cover several pathological conditions and, consequently, and could therefore help improve clinical decision making.

## Conclusion

The present study proposed and evaluated a filter approach for feature selection in classification using an evaluation function, the $\gamma$-metric. This approach yielded encouraging results for its application in AF detection. Indeed, AF is frequently paroxysmal and/or asymptomatic; moreover, its prevalence increases with age and it is one of the leading causes of stroke. Developing efficient automated tools for early AF detection could help physicians better manage this disorder, including via the administration of oral anticoagulation treatment which has proven to be highly efficacious for stroke prevention. In this perspective, feature selection combined with classification could offer new strategies for quasi real-time diagnosis using other types of big data, in particular physiological data obtained with connected health objects and mobile health applications.

## Supplementary Information

**Additional file 1.** Step-by-step mathematical derivation of Equation 4 to compute *d*_*k*1_, _*k*1*k*2_.

## Data Availability

Data from the Physionet website are in open access. To obtain 24-hours Holter data from the Department of Cardiology and Rhythmology of Marseille University Hospital Center (Timone Hospital), researchers can submit a written request to roch.giorgi@ap-hm.fr.

## Abbreviations

AF: Atrial fibrillation
afdb: Atrial fibrillation database
AUC: Area under the curve
CART: Classification and regression tree
ECG: Electrocardiogram
GA: Genetic algorithm
HRV.index: Heart rhythm variability index
IRRR: Interquartile range of RR intervals
KI: Kuncheva index
MADRR: Median of the absolute differences between adjacent RR intervals
MCC: Matthews correlation coefficient
MDA: Mean decrease in accuracy
MDG: Mean decrease in Gini index
MIT-BIH: Massachusetts Institute of Technology-Beth Israel Hospital
NSR: Normal sinus rhythm
nsrdb: Normal sinus rhythm database
pNN50: Percentage of differences between successive RR intervals greater than 50 ms
RF: Random forest
RMSSD: Root-mean-square of successive differences
ROC: Receiver operating characteristic
SDANN: Standard deviation of the averages of 5-s RR intervals
SDNN: Standard deviation of all RR intervals
SDNNidx: Mean of all standard deviations of 5-s RR intervals
SDSD: Standard deviation of successive differences
SMOTE: Synthetic minority over-sampling technique
SVM: Support vector machine
TINN: Triangular interpolation of the RR interval histogram
