# Supplementary material for: A filter approach for feature selection in classification: application to automatic atrial fibrillation detection in electrocardiogram recordings
Source: BMC Med Inform Decis Mak. 2021 May 4;21(Suppl 4):130. doi: 10.1186/s12911-021-01427-8 (PMC8094578; doi:10.1186/s12911-021-01427-8)
Supplement: Supplementary file 1 — Additional file 1. Step-by-step mathematical derivation of Equation 4 to compute dk1, k1k2. [file 12911_2021_1427_MOESM1_ESM.pdf]

Additional file 1 - Step-by-step mathematical derivation of d_{k_1,k_1k_2} and d_{k_2,k_1k_2}

Pierre Michel, Nicolas Ngo, Jean-François Pons,
Stéphane Delliaux and Roch Giorgi

December 16, 2020

We propose a step-by-step mathematical derivation of Equation 4 to compute d_{k_1,k_1k_2}, which is the distance between the centroid of ellipse k_1 and any point on its border in the direction of the vector towards the centroid of another ellipse k_2. We detail this calculation only for d_{k_1,k_1k_2}, as the calculation for d_{k_2,k_1k_2} is identical by symmetry (where k_1 is replaced by k_2).

Let us recall that ellipse k_1 centered on $\boldsymbol{\mu_{k_1}} \in \mathbb{R}^p$ is defined by p axes which correspond to the normalized eigenvectors $\{\boldsymbol{u_{k_1,j}}\}_{j=1,...,p}$ of the covariance matrix $\boldsymbol{W_{k_1,p}}$, of length $\sqrt{\lambda_{k_1,j}}$, where $\{\lambda_{k_1,j}\}_{j=1,...,p}$ are the eigenvalues of $\boldsymbol{W_{k_1,p}}$. The mean-mean vector is given by $\boldsymbol{\mu_{k_1}\mu_{k_2}} = \boldsymbol{\mu_{k_2}} - \boldsymbol{\mu_{k_1}}$, where $\boldsymbol{\mu_{k_2}} \in \mathbb{R}^p$ is the centroid of ellipse k_2.

Let $\boldsymbol{\tilde{\mu}_{k_1}} \in \mathbb{R}^p$ be the vector of coordinates $\tilde{\mu}_{k_1,j}, j = 1, ..., p$. Each coordinate then represents the orthogonal projection of the normalized mean-mean vector on an eigenvector, as follows:

$$\tilde{\mu}_{k_1,j} = \left\langle \frac{\boldsymbol{\mu_{k_1}\mu_{k_2}}}{||\boldsymbol{\mu_{k_1}\mu_{k_2}}||}, \boldsymbol{u_{k_1,j}} \right\rangle,$$

where $\langle , \rangle$ is the scalar product. See Equation 5 for the expression of vector $\boldsymbol{\tilde{\mu}_{k_1}}$ in matrix formulation.

Let $\boldsymbol{D} \in \mathbb{R}^{p\times p}$ be the diagonal matrix containing the values $\{\sqrt{\lambda_{k_1,j}}\}_{j=1,...,p}$, we define $\boldsymbol{v_{k_1}} \in \mathbb{R}^p$ as follows:

$$\boldsymbol{v_{k_1}} = \boldsymbol{D}^{-1}\boldsymbol{\tilde{\mu}_{k_1}}.$$

This corresponds to the linear application that moves the ellipse into the unit circle. Since the unit vector given by the cluster centroids is transformed

by this application, we can write:

$$||\boldsymbol{v_{k_1}}|| = \sqrt{\sum_{j=1}^{p} \frac{\tilde{\mu}_{k_1,j}^2}{\lambda_{k_1,j}}}.$$

We notice that in the unit circle, the distance between μ_{k_1} and any point on the border of the circle is 1. We can therefore write the ratio between this distance and the norm of the transformed mean-mean vector.

Recalling that a linear application preserves the ratios between lengths, we can define the distance d_{k_1,k_1k_2} by inverse application (whereby the unit circle is transformed into the ellipse) as follows:

$$d_{k_1,k_1k_2} = \frac{1}{||\boldsymbol{v_{k_1}}||} = \frac{1}{\sqrt{\sum_{j=1}^{p} \frac{\tilde{\mu}_{k_1,j}^2}{\lambda_{k_1,j}}}}.$$

By symmetry, we have:

$$d_{k_2,k_1k_2} = \frac{1}{||\boldsymbol{v_{k_2}}||} = \frac{1}{\sqrt{\sum_{j=1}^{p} \frac{\tilde{\mu}_{k_2,j}^2}{\lambda_{k_2,j}}}}.$$
